# Modifiable and Non-Modifiable Factors That Affect Human Milk Oligosaccharides Composition

**DOI:** 10.3390/nu16172887

**Published:** 2024-08-28

**Authors:** Małgorzata Konieczna, Anna Koryszewska-Bagińska, Agnieszka Bzikowska-Jura, Magdalena Chmielewska-Jeznach, Sylwia Jarzynka, Gabriela Olędzka

**Affiliations:** 1 Department of Medical Biology, Medical University of Warsaw, 00-575 Warsaw, Poland; 2 Laboratory of Human Milk and Lactation Research, Department of Medical Biology, Medical University of Warsaw, 00-575 Warsaw, Poland

**Keywords:** human milk oligosaccharides, infants, modifiable and non-modifiable factors, maternal factors, nutrition

## Abstract

Human milk, the gold standard in infant nutrition, is a unique fluid that provides essential nutrients such as lactose, lipids, proteins, and free oligosaccharides. While its primary role is nutritional, it also protects against pathogens. This protection mainly comes from immunoglobulins, with human milk oligosaccharides (HMOs) providing additional support by inhibiting pathogen binding to host cell ligands. The prebiotic and immune-modulatory activity of HMOs strongly depends on their structure. Over 200 individual structures have been identified so far, with the composition varying significantly among women. The structure and composition of HMOs are influenced by factors such as the Lewis blood group, secretor status, and the duration of nursing. HMO profiles are heavily influenced by maternal phenotypes, which are defined based on the expression of two specific fucosyltransferases. However, recent data have shown that HMO content can be modified by various factors, both changeable and unchangeable, including diet, maternal age, gestational age, mode of delivery, breastfeeding frequency, and race. The first part of this overview presents the historical background of these sugars and the efforts by scientists to extract them using the latest chromatography methods. The second part is divided into subchapters that examine modifiable and non-modifiable factors, reviewing the most recent articles on HMO composition variations due to specific reasons and summarizing potential future challenges in conducting these types of studies.

## 1. Introduction

Human milk oligosaccharides (HMOs) have been an enigmatic and captivating field of scientific research for over a century, spreading curiosity among researchers and healthcare professionals. The history of HMO research dates back to the late 19th century, when early scientists made intriguing observations regarding the correlation between infants’ gut health and breastfeeding. Pioneering researchers, such as Theodor Escherich in 1886, drew attention to the presence of intestinal bacteria in the feces of breastfed infants and their physiology of digestion. He postulated a relationship between the infant’s gut microbiota and the properties of breast milk [1], which was subsequently continued by Moro and Tissier [2]. While the specificity of HMOs had not been discovered, these early observations laid the groundwork for future investigations into the unique components of human milk. In the mid-20th century, scientific progress in analytical techniques and molecular biology set the stage for deeper research into these molecules and/or components of breast milk. Scientists began to characterize the non-lactose carbohydrates present in breast milk, which were distinct from those found in bovine milk. They later called them “gynolactose” [3,4]. Nevertheless, both the exact structure and functions were still unknown.

Further research led to the discovery of a factor in human milk that promoted the growth of *Bifidobacterium bifidus* (previously known as *Lactobacillus bifidus*) [4]. This discovery spurred a series of experiments that allowed scientists to characterize the chemical composition of this factor [5,6,7]. In the following years, fourteen oligosaccharides were found in human milk. All of them contained lactose and unexpectedly had an activity with blood group determinants, which was the next step towards their characterization [8,9].

With the advancement of chromatographic techniques, such as thin-layer chromatography (TLC) and high-performance liquid chromatography (HPLC), more than 200 oligosaccharides have been discovered and characterized in human milk. This enabled researchers to identify diversity, both in composition and function [10,11,12].

As research progressed, the biological significance of HMOs became increasingly significant. Certain HMOs, such as 2’-fucosyllactose (2’-FL) and lacto-*N*-neotetraose (LNnT), have been found to selectively promote the growth of beneficial intestinal bacteria, especially *Bifidobacteria*, while inhibiting the growth of harmful pathogens [13,14,15,16,17]. Additionally, HMOs exhibited anti-adhesive properties, which effectively prevented the attachment of pathogenic bacteria, such as *Escherichia coli* and *Salmonella fyris,* to intestinal epithelial cells, thereby reducing the risk of infection [18,19].

Moreover, an increasing number of studies on the immunomodulatory effects of HMOs have shown that their positive impact on the maturation and functioning of the infant’s immune system contributes to the development of a balanced and strong immune response [20,21,22].

Recent research has also focused on the potential impact of HMOs on brain development and cognitive functions. Studies in animal models suggest that HMOs may influence neural development and cognitive function, although the exact mechanisms are still unknown [23,24].

Additionally, longitudinal studies have been conducted to examine the potential long-term health effects associated with HMO consumption during infancy, which have shown that HMO consumption reduces the risk of certain infections, allergies, and chronic diseases later in life [22,25].

HMOs have gained considerable recognition in the field of chemistry due to their complex nature. HMOs are the third most abundant component of breast milk, after lactose and fats. Remarkably, they exhibit an average concentration of 12.9 g/L in mature milk and 20.9 g/L 4 days postpartum. These complex carbohydrates are primarily constructed by 5 monosaccharides: D-glucose (Glc), D-galactose (Gla), *N*-acetylglucosamine (GlcNAc), L-fucose (Fuc), and sialic acid (Neu5Ac). The framework of HMO structure is lactose that can be additionally sialylated (e.g., 3′sialyllactose, 3’SL) or fucosylated (e.g., 2’-fucosyllactose). These bonds, formed by various possible elongations, are responsible for the formation of over 200 different types of oligosaccharides. The variety and configuration of these attached sugar moieties determine their chemical and biological properties, leading to the delineation of three distinct categories of HMOs: neutral, sialylated, and fucosylated [26,27,28].

The discovery and subsequent characterization of HMOs in human milk prompted researchers to investigate their relationship to blood group antigens, thus opening a new direction in the study of these compounds/molecules. Interestingly, they have shown that not every woman synthesizes the same set of oligosaccharides. This structural diversity of HMOs is due to the fact that they are synthesized by the mother’s mammary gland, which is influenced by many factors including maternal diet, genetics, and lactation stage.

The composition of HMOs varies greatly among individuals and populations, and different types and amounts of HMOs present in breast milk vary depending on these factors, not all of which can be modified. Understanding the complex composition of HMOs and the factors influencing their variability is essential for comprehending their diverse roles in infant health and development. Previous overviews on HMO composition may have overlooked key information. In this overview, we explore the multifaceted determinants that shape the composition of HMOs. Our goal is to shed light on the intricate interplay of maternal, environmental, and genetic factors contributing to the dynamic nature of HMOs in human breast milk. Through this exploration, we seek to gain insight into the mechanisms underlying HMO diversity.

## 2. Non-Modifiable Factors

### 2.1. Genetic Background

Regarding the composition of HMOs, maternal genetic factors appear to be the main cause of their wide and unique variability. The biosynthesis of HMOs and their structure is primarily influenced by the activity of two genes, namely FUT2 and FUT3, which encode α-1-2 and α-1-3/4 fucosyltransferase, respectively [28,29,30]. The different expression of these two genes affects the Lewis blood group (Le) and maternal secretor status (Se). More specifically, the Lewis blood group system is regulated by the FUT3 gene, while the FUT2 gene activity is responsible for the secretion of these antigens into body fluids, including breast milk [29]. Four distinct maternal phenotypes can be identified based on the expression of these genes, leading to different milk groups with diverse HMO compositions [30].

The Se+/Le+ (a−b+) phenotype, associated with the expression of both genes, is referred to as a secretor. The Se−/Le+ (a+b−) phenotype, determined by the expression of only the FUT3 gene, characterizes non-secretors. Lack of FUT3 gene expression results in the Le− (a−b−) phenotype, where both secretors (Se+/Le−) and non-secretors (Se−/Le−) can be found [28,31]. The frequency of specific blood group phenotypes associated with the Lewis system is as follows: Le (a−b+) 72%, Le (a+b−) 22%, Le (a−b−) 6% [29]. The activity of the FUT2 and FUT3 genes is also responsible for different linking attachments. For example, FUT2 connects fucose to lactose, creating 2’-FL, or to lacto-*N*-tetraose (LNT), producing lacto-*N*-neotetraose (LNFP I). On the other hand, the FUT3 gene, by adding more fucoses, produces lacto-*N*-difucohexaose I (LDFH I), lactodifucotetraose (LDFT), lacto-*N*-neotetraose II (LNFP II), 3FL, and lacto-*N*-neotetraose III (LNFP III) [28,32]. The FUT2 and FUT3 genes are highly polymorphic, and single nucleotide polymorphisms (SNPs) identified within both genes determine the enzymatic activity of the encoded fucosyltransferases in various populations. Women who are homozygous for a non-functional variant of the FUT2 gene, known as the secretor gene, are unable to produce HMOs containing the fucose moiety. This results in lower levels of certain fucosylated HMOs in their breastmilk, including 2’-fucosyllactose (2’-FL) and lacto-*N*-fucopentaose I (LNFP I) [32,33,34,35]. Conversely, women with specific variants of the FUT3 gene have been found to produce higher levels of HMOs containing the Lewis a and Lewis b antigens, which are also fucosylated. These variants are associated with increased levels of HMOs such as 3-fucosyllactose (3-FL) and Lewis b-containing HMOs [33,36]. However, variability in certain genetic polymorphisms may lead to distinct outcomes. For instance, even in the absence of active FUT3 gene function, trace amounts of 2’-fucosyllactose (2-’FL) are detected. Similarly, compounds like 3’-fucosyllactose (3’-FL) and LNFP III found in Lewis-negative (Le−) individuals might exhibit a correlation with the functional output of alternative fucosyltransferase genes [36,37].

There are four milk groups determined by different oligos based on the expression of the FUT2 and FUT3 genes. The first group is Se+/Le+ (a−b+) and has the most diverse composition, containing 2-‘FL, 3’-FL, DFL, LNT, LNnT, LNFP I, LNFP II, LDFH I, and LDFH II. The second group is Se−/Le+ (a+b−) and has a simpler composition, containing 3’-FL, LNT, LNnT, LNFP II, LNFP II, and LDFH III. The third group is Se+/Le− (a−b−) and contains 2-‘FL, 3’-FL, DFL, LNT, LNnT, LNFP I, and LNFP III [38]. The fourth and last phenotype, Se−/Le−, is rare and has no activity for both FUT2 and FUT3 genes. Consequently, six main oligosaccharides present in other milk groups should not be observed. Wang et al.’s research on the Le− phenotype revealed the absence or undetectable levels of the six possible HMOs in milk samples, or their levels were undetectable. In Williams’ study [33], they confirmed, as previously mentioned [32], that the amounts of 2’-FL are entirely correlated with polymorphisms in FUT2. This correlation allows the prediction of secretors and, conversely, the prediction of 2’-FL levels based on secretor status.

However, polymorphisms around FUT3 are more complex than in the FUT2 gene. Thus, in mother’s milk, which was Lewis positive, LNFP II was absent [32]. The authors assume that Lewis status depends on more genetic variations in the FUT3 gene, which are still unknown. It is also likely that polymorphisms in FUT genes vary between populations, impacting HMO composition [32,33,39,40,41,42].

### 2.2. Race/Ethnicity

**Figure 1:**
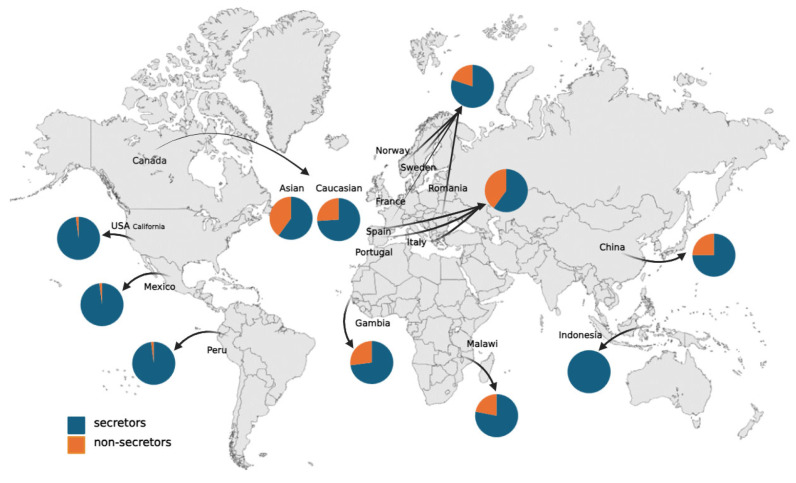
Visual distribution of secretor and non-secretor phenotype within world population, according to the race/ethnicity factor. The figure was created using BioRender (www.biorender.com), (accessed on 21 August 2024).

What is most concerning about HMOs is that they change their proportion and amount not only according to the Lewis phenotype and secretor status but also to the mother’s nationality. Surprisingly, the secretor status frequency also differs geographically, as visualized in various populations across the world (Figure 1). Depending on genetic variations, secretors/non-secretors are supposed to contribute 80/20%, respectively. However, ethnicity can alter this differentiation. The McGuire et al. group [43] made a study on seven countries’ populations, where 98% of secretors were found in Peru and the USA—California state, where they identify themselves as Hispanic. The same conclusions were found for the Mexican population [36,44]. Consequently, with almost only secretors in the population, HMO diversity was less than in other countries. A study conducted on the Indonesian cohort also uncovered only the secretor group (with 120 participants) [45]. In contrast, the Caucasian population is distributed into ~80% of secretors [37,43,46,47], while Asian ethnicity covers only ~60% of secretors versus 40% of non-secretors, which is unattainable in Caucasian [43,47,48,49]. African mothers were 70–80% secretors [50,51] Within European countries, the secretors frequency was not equal, representing Sweden, France, Romania, and Norway at 70–90%, while in Portugal, Spain, and Italy it was just 50–60% of the population [46], which divides them geographically (Figure 1). In the non-secretors group, the Lewis-negative phenotype in the white population is extremely rare (1–6%) [28,52], compared to certain Latin American and African countries with approximately 30% [53,54].

In the composition of HMOs, variations were observed across different countries. According to McGuire et al. studies [43], Swedish mothers exhibited higher amounts of 3’-FL in their milk compared to African mothers. Milk produced by mothers from Peru was highly abundant in 2’-FL, 3’-FL, and 3’-SL, where the frequency of secretors was 98%. LNFPIII showed remarkably high concentrations in Sweden as well, in comparison to Gambia, Ethiopia, even the USA and Spain. Similar conclusions were made by the Erney group [36], where samples from Europe and the United States provided similar means for LNFPIII, as did those from Asia and Latin America; however, they did not find any meaningful differences of 3’-FL and LNFPIII concentrations between nations. These findings contrast with a study conducted by the Gomez-Gallego group [55], which reported that Finnish mothers had milk with lower 3’-FL concentrations than their African counterparts. Interestingly, apart from 3’-FL amounts, Chinese and South African mothers had milk with higher levels of LNFPIII than Finnish mothers, while 2’-FL and LNFPI amounts were lower. However, the answer to those differences is in the background knowledge of secretor status frequency that appears in these populations. 2’-FL consistently appears alongside secretor status. In cases where 2’-FL levels are low, the second most prevalent oligosaccharide, 3’-FL, may indicate non-secretor status. Additionally, 3’-FL levels can vary significantly between nations, potentially highlighting notable differences.

While specific HMO levels did not exhibit such significant differences between countries, other groupings of HMOs, based on a modification—fucosylation, sjalylation—revealed variations among different cohorts. Swedish and USA mothers had milk more fucosylated than sialylated. Sialylated oligosaccharides, like LSTc, appeared in higher amounts in Asian mothers compared with Caucasian mothers [47], while LSTb was lower in Hispanic ethnicity [43]. On the contrary, it is noteworthy that the milk from non-secretor mothers in Malawi and Gambia exhibited reduced levels of both fucosylated and sialylated HMOs [50,51]. Including mother’s diet and geographic location, this observation is particularly significant, as it may have implications for various health conditions affecting infants in this region.

This diversity among ethnic groups likely stems from evolutionary forces that either favor or discourage fucosylated HMOs, potentially leading to structure-specific impacts on infant development and susceptibility to diseases.

### 2.3. Lactational Stage

To date, numerous studies have demonstrated that the composition of human milk oligosaccharides changes throughout the lactation period, and several overviews came out [56,57]. These changes are significant, with HMO composition decreasing over the 20-week lactation period, regardless of the mother’s Lewis and secretor status. In this subchapter, we considered including the newest articles and propose some summarization of the most important changes and varieties through milk composition.

Research indicates that breast milk can be categorized into different stages, each characterized by unique HMO compositions. Broadly, breast milk can be divided into stages: colostrum, the initial liquid produced after delivery and lasting for about 4 days; transitional milk, occurring from 5 to 14 days postpartum; and mature milk, considered largely mature up to 30 days postpartum. The final stage of lactation is fully mature milk [58]. In colostrum, the highest levels of HMOs are found. Among secretors, fucosylated [59,60,61] or in some cases specifically 2’-FL [36,45,47,49,62,63,64,65,66,67] are the most abundant oligo and remain stable for over 30 days postpartum; however, on the first day postpartum, they were significantly higher than day 2 [68]. Throughout lactation, 2’-FL levels generally decrease along with 3’-FL increase, whether it was the secretor or non-secretor group. Notably, the key difference between secretors and non-secretors is that secretors initially have higher amounts of 2’-FL than 3’-FL, whereas non-secretors have higher levels of 3’-FL than 2’-FL from the beginning of lactation [36,45,63,65,67,69,70]. In non-secretors, LNT is predominant in colostrum and transitional milk [49,62,71], while 3’-FL is higher in mature milk [71]. After 4 months of lactation, LNT and LNnT significantly decreased in both groups [62,63,64,65,66]. However, Asher et al. concluded that in secretors, LNT increased over time. LNFPI and II remain stable over the lactation, certainly up to one month of lactation, then LNFPI and III decrease, while LNFPII increases over time, up to 4 months. After that time, LNFPII also gradually decreased [36,49,63,66,71].

Among sialylated oligosaccharides, 3’-SL is the most abundant on the first day postpartum, whereas its levels were lower on days 2 and 3. Conversely, 6’-SL levels were high on day 3 and lower on day 1. From the colostrum stage to mature milk, 3’-SL levels decrease over time in both secretors and non-secretors [49,64,69,71,72]. In contrast, several studies have reported stable levels of 3’-SL over 4 months of lactation [32,49,62,64,65], while other studies demonstrated increasing levels of 3’-SL up to 4 months of lactation [47,63,66]. Beyond this period, the overall amount of 3’-SL tends to increase [32,47,65]. Similarly, levels of DSLNT [63,71] and 6’-SL [32,49,62,64,65,66] also decreased over time; however, two research groups observed a different pattern where 6’-SL increased from colostrum to transitional milk before decreasing, contrasting with other oligosaccharides [49,69]. As long as lactation extends, LSTc oligosaccharide decreases as well [32,49,65,66,69].

While several studies have indicated a significant decrease in oligosaccharide levels over a 20-week lactation period, one study presents contrasting observations. According to this study, milk composition checked at 6 weeks and 16 weeks postpartum showed no significant changes in HMO composition [73].

### 2.4. Mother’s Age

While investigating the impact of maternal age on oligosaccharide content, only several studies have been conducted. Maternal age was negatively associated with LNnT, LST, DSLNH [43,74], and DFLNT [47] amounts, while a positive correlation was observed with FLNH [43], 2′-FL, and DFLNH-b [74].

However, when considering additional factors such as ethnicity, BMI, time postpartum, and parity, there is insufficient evidence to strongly support the notion that maternal age significantly influences overall HMO concentrations [43]. In this context, it becomes crucial to explore alternative perspectives. According to studies conducted by McGuire, the conclusion drawn is that certain oligosaccharides exhibit a varying pattern when examined from different angles. Despite an apparent decrease in the overall number of oligosaccharides in older women, the quantities of FLNH and DFLNH become noteworthy as women age [43,63].

It is essential to acknowledge that as maternal parity increases, there is an associated rise in age. When considering the impact of both parity and age on HMO concentrations, it becomes evident that the levels of LNnT oligosaccharides in breast milk tend to decrease [43,46,74] which can also have a similar effect in mothers’ BMI.

### 2.5. Parity

To date, few studies have investigated associations between parity and concentrations of HMOs, and existing literature lacks consensus on the matter. Some research has focused on first-time mothers (primiparous) and identified elevated levels of certain HMOs, such as LNFP III [43], 2’-fucosyllactose (2’-FL), coupled with lower concentrations of lacto*-N-*neotetraose (LNnT) [43,47]. Conversely, other studies have shown varied correlations, highlighting significantly higher concentrations of DSLNT (at day 2) and LNnT (at day 17) among primiparous women. Additionally, these studies have reported notably lower levels of 6′GL and 3FL (at day 2) and LNFP II and LNFP V (at day 17) among first-time mothers [46]. This association is observed in both secretor-positive and secretor-negative mothers.

For multiparous mothers, who have experienced multiple childbirths, the HMO profiles in their breast milk may exhibit differences compared to primiparous mothers. Examples include slightly lower concentrations of 3’-FL but higher levels of LNnT [47,75]. However, contrasting findings by Richardson et al. suggest an overall increase in total HMOs with parity.

On the other hand, Elwakiel et al. [61] did not find any significant links between parity and HMO composition in their studies. Nevertheless, it remains challenging to establish a definitive correlation between maternal parity and the composition of HMOs, as the evidence in this area is inconclusive.

## 3. Modifiable Factors

### 3.1. Mode of Delivery

Natural vaginal delivery is considered to have more positive impacts on child health than cesarean section. In natural vaginal delivery, the baby passes through the birth canal, coming into contact with the mother’s vaginal and fecal microbiota. This exposure is thought to contribute to the initial colonization of the infant’s gut with beneficial bacteria. Human milk composition is also changing via natural delivery when it comes to increasing levels of nutrients. Some previous studies suggest that there is no discernible connection between the concentration of HMOs and the mode of delivery [48,75], whether mothers are secretors or non-secretors [75,76]. Another study also proposed that there is no clear link between the mode of delivery and the composition of HMOs [47].

However, a recent study conducted by Samuel et al. [77] found that there are noteworthy differences in HMO concentrations between women who delivered via C-section (CS) and those who had vaginal deliveries. Specifically, after adjusting to milk groups, mothers who underwent C-sections had significantly lower levels of 2-’FL, 3-’SL, and 6-’GL in their breast milk. Mothers who delivered naturally also exhibited elevated levels of LNFPIII, as indicated by studies conducted by the Gomez-Gallego group [55]. The authors identified notable variations among mothers who delivered via C-section from different countries; however, it is important to note that the data was not adjusted for milk groups in the analysis.

Nevertheless, it should be noted that a definitive explanation for these differences is still unknown. The process of undergoing a C-section can be emotionally and physically stressful for mothers, potentially leading to alterations in stress hormone levels. These changes may then affect the composition of HMOs in breast milk. Further research is needed to explore this relationship using different research approaches, as it has not been thoroughly investigated before.

### 3.2. Gestational Age

Premature infants often face challenges like respiratory issues, jaundice, feeding difficulties, and developmental delays due to their underdeveloped organs. Studies have shown that LNnT is more abundant in the milk of women delivering preterm. Furthermore, preterm milk oligosaccharide fucosylation is not well regulated, which results in immaturity of HMO production and temporary absence of 2’-FL [78]. That may have relevance to the susceptibility of premature infants to necrotizing enterocolitis.

However, other studies disagreed and showed no significant differences between preterm and term milk in neutral or acidic fractions of HMOs, the same as in overall HMO amounts [47,79,80]. Austin et al. [81] have found increased amounts of sialylated HMOs, 3′SL, LSTb, and DSLNT, in preterm milk, which can be the explanation for protecting infants from the development of NEC, as it was proven on rats [82]. Mothers of preterm infants frequently confront impediments to breastfeeding attributable to limitations in milk production. Nevertheless, the utilization of milk obtained from a milk bank for the nourishment of preterm neonates has demonstrated a consequential reduction in complications, notably the incidence of necrotizing enterocolitis.

The studies had certain limitations as the researchers did not categorize human milk oligosaccharides (HMOs) into milk groups based on secretory status, except for the Wang group, which performed this classification. Wang’s group identified significant effects in both non-secretor and secretor groups. According to their findings, gestational age exhibited a negative association with IFLNH-I and TFLNH-II in secretors, and with 3′-FL, LNFP-II, LNFP-IV, DFpLNH-I, and DFpLNH-II in non-secretors [74].

### 3.3. Breastfeeding Frequency and Duration

Although there are no direct studies linking breastfeeding frequency to the composition of HMOs, frequent breastfeeding, especially during the first few months after childbirth, is crucial in providing infants with essential nutrients. It is noteworthy that the highest concentration of oligosaccharides is usually present in a mother’s milk during the first month after childbirth [83]. Furthermore, frequent breastfeeding not only stimulates milk production but also increases the overall volume of milk a mother produces, which may lead to a higher concentration of HMOs in milk. Additionally, frequent breastfeeding may play a role in maintaining the balance of the infant’s gut microbiota, which in turn can influence HMO composition [84,85].

Research conducted by Azad et al. [47] reveals a positive correlation between breastfeeding duration and the increase in Lacto*-N-*tetraose (LNT) levels. Each additional month of breastfeeding contributes to this increase. This correlation holds independently of factors such as the duration of lactation, the exclusivity of breastfeeding at the time of milk sample collection, and other possible variables. Furthermore, an extended duration of breastfeeding was also associated with higher levels of FDSLNH, while an inverse relationship was observed with 3’SL levels.

### 3.4. Maternal Diet

Exploring the relationship between maternal dietary intake and human milk oligosaccharide composition presents a challenge due to the complexity of this interplay. The limited number of studies addressing this topic underscores the methodological difficulties inherent in such investigations. To advance understanding in this area, we advocate for a review of the existing literature, which may provide valuable insights into the intricate dynamics of maternal nutrition and breast milk composition. While genetics undoubtedly plays a role in determining the different types of HMOs a mother produces, emerging evidence suggests that dietary choices can also influence the abundance and diversity of specific HMOs. This impact assumes a critical role in certain instances, as evidenced by the diminished levels of sialylated milk oligosaccharides observed in mothers from Malawi with undernourished infants. To investigate this phenomenon, researchers introduced bacterial strains sourced from the gut microbiota of malnourished Malawian infants into young germ-free mice. Supplementing a typical Malawian diet with purified sialylated bovine milk oligosaccharides (S-BMOs) resulted in enhancements in lean body mass, alterations in bone structure, and changes in metabolic processes in the liver, muscles, and brain. These changes implied improved nutrient utilization to support growth [50].

Dietary macronutrient composition plays a pivotal role in HMO modulation, with a lipid-rich diet, as opposed to a carbohydrate-focused one, correlating with diminished levels of sialylated HMOs. Similarly, the selection between glucose and galactose in the diet substantially influences the concentrations of fucosylated HMOs [85,86,87]. However, there was no significant difference in individual HMOs between these dietary regimens [85]. The levels of sialylated HMOs show a significant connection with the composition of the microbiome in both dietary groups. This suggests that alterations in sialylated HMO concentrations may be linked to variations in bacterial proliferation within the milk microbiome, potentially influenced by maternal dietary choices impacting HMOs [86]. These oligosaccharides remain indigestible by infants, but they are beneficial to bacteria [85]. Furthermore, the consumption of probiotics has a link to the variations in some HMO concentrations [88].

Azad et al.’s investigations revealed associations between specific HMOs and dietary components [47]. Fucosyllacto*-N-*hexaose exhibited a positive correlation with whole grain consumption, while LSTb demonstrated a negative correlation with total protein and empty calorie intake. Additionally, LNT and difucosyllacto*-N-*hexaose are positively related to total energy intake. Nevertheless, a more comprehensive analysis reveals that only lactational stage and secretor status are independently linked to the overall concentration of HMOs. Furthermore, it was found that mothers who consumed multivitamins had higher levels of DSLNH [47]. Notably, vitamin B supplementation positively correlated with increased levels of 2’-FL and 3’-FL, and higher intake of vitamin A was associated with increased levels of 3’-FL [89] and sialylated HMOs [90,91,92]. Moreover, the consumption of vegetables was linked to higher levels of 3’-FL, while fruit intake was correlated with increased sialylated HMOs [90]. Conversely, high-sugar and high-fat food consumption was linked to decreased HMO quantities [89]. The Jorgensen et al. group, in comparison, could not observe significant differences in HMO composition in mothers who were consuming micro- and macronutrient supplements [93]. It is important to note that the studies had certain limitations, including a limited number of factors analyzed.

The study undertaken by Neville et al. [94] explored potential distinctions in HMOs among mothers following vegan, vegetarian, and non-vegetarian diets, and concluded that women who consume plant-based diets do not produce different breast milk as it relates to HMO composition. On the contrary, a study conducted by Bottin et al. [95] showed that HMOs increased levels were associated with higher consumption of meat and poultry. Notably, diet exerted a more pronounced impact on HMO content in secretor mothers than in non-secretor mothers [87,90]. It is important to recognize that while diet contributes to HMO composition, its overall significance may vary among individuals. Genetic, hormonal, BMI, geography, and other health-related factors combine to influence HMO profiles. It is important to understand that the relationship between diet and HMO composition is complex, and ongoing research is still trying to uncover the precise details. Therefore, to optimize breast milk composition, mothers should maintain a balanced and nutritious diet that includes a variety of whole foods and supports their overall health and lactation.

### 3.5. Maternal ppBMI

Interestingly, not only the mother’s diet but also the maternal body mass index (BMI) can affect the concentrations of HMOs in breast milk. It has been observed that obese mothers tend to exhibit a heightened reluctance towards breastfeeding [96].

Some studies have found that maternal weight and BMI are positively correlated with 2′-fucosyllactose [43,61,75,97], fucosylated HMO [43,98], total HMO [61,98], and maternal weight was positively correlated with LNFP III and DFLNT [43,75] in human milk. Conversely, maternal weight and BMI were inversely correlated with LNnT [43,46,63,69,97], LNH [47], and DSLNT [43,69].

However, some other research has failed to find a direct correlation between BMI and HMO composition [47,92,99]. This discrepancy may be attributed to other factors, such as prolactin levels. A group of researchers conducted a study to investigate how prolactin affects breast milk composition. Previous research indicated that mothers with preterm infants or those who are obese usually have lower levels of prolactin. The study found that administering human prolactin (r-hPRL) increased the levels of both neutral and acidic oligosaccharides, indicating that prolactin may help synthesize certain carbohydrates during lactation. These findings suggest that prolactin plays a role in enhancing the quality and quantity of breast milk [100].

## 4. Summary

Human milk stands as the gold standard for newborns, offering not only a rich source of nutrients but also numerous bioactive components with potential therapeutic effects [101]. Among these, human milk oligosaccharides (HMOs) emerge as key players, captivating scientists for over a century. Originating from historical observations linking infant gut health to breastfeeding, the journey of HMO exploration has led to modern research unveiling their diverse functions, including fostering beneficial gut bacteria growth, deterring pathogen attachment, and modulating the immune system. This overview scrutinized factors influencing human milk composition, particularly HMO content. Maternal genetic factors, notably the activity of *FUT2* and *FUT3* genes encoding fucosyltransferases, significantly influence HMO variability and maternal secretor status, thereby impacting infant health and development. However, recent research emphasizes that genetic factors alone do not dictate human milk composition. Geographic variations in *FUT2* and *FUT3* gene polymorphisms, coupled with shifts in lactational stages, contribute to fluctuations in oligosaccharide content. Additionally, while factors like diet, gestational stage, and maternal age may influence HMO composition, their effects may not always be statistically significant, as revealed by subsequent multivariable analyses. A summary of these associations is presented in the table below (Table 1). Understanding these influences is imperative for optimizing infant health outcomes and guiding future research endeavors. Future studies should consider targeting specific populations and stratifying them based on secretor status and lactational stage to ensure robust conclusions. Nonetheless, such stratification may constrain the generalizability of findings due to group size limitations. Despite this challenge, comprehending these factors is crucial for developing interventions aimed at preventing conditions such as necrotizing enterocolitis (NEC) and safeguarding newborns through targeted supplementation.

## Figures and Tables

**Table 1:** Summary of the reported associations between oligosaccharides and both maternal and infant factors.

Oligosaccharide *	Factor	Association	References
2’-FL	genetic	Absent or very low amounts in non-secretors, presence correlated with FUT2 gene	[32,33,34,35]
	race/ethnicity	↑ in milk from Peruvian mothers	[43,44]
	lactational stage	↓ over lactation	[36,45,63,65,67,69,70]
	mother age	↑ with age	[43,74]
	parity	↑ in primiparous mothers	[43,46,47]
	mode of delivery	↓ in C-section deliveries	[55,77]
	maternal diet	↑ with vitamin B intake	[89]
3’-FL	genetic	↑ with certain FUT3 gene variants, found in Lewis-negative (le-) individuals	[33,36] [36,37]
	race/ethnicity	↑ levels in milk from Peruvian mothers, ↑ in Asian mothers	[43,44] [47]
	lactational stage	↑ over lactation	[36,45,63,65,67,69,70]
	parity	↓ in multiparous mothers	[43,46,47]
	gestational age	negative association with gestational age	[74]
3’-SL	race/ethnicity	↑ levels in milk from Peruvian mothers	[43,44]
	lactational stage	↑ after 4 months of lactation, before that time levels debatable	[32,47,65]
	mode of delivery	↓ in C-section deliveries	[55,77]
	breastfeeding frequency	↓ with breastfeeding duration	[47,84,85]
6’-SL	lactational stage	↓ over lactation	[32,49,62,64,65,66]
6’-GL	mode of delivery	↓ in C-section deliveries	[55,77]
LNT	lactational stage	↓ after 4 months of lactation	[62,63,64,65,66]
	breastfeeding frequency	↑ with breastfeeding duration	[47,84,85]
LNnT	lactational stage	↓ after 4 months of lactation	[62,63,64,65,66]
	mother age	↓ with age	[43,47,74]
	parity	↓ in multiparous mothers	[43,46,47]
	gestational age	more abundant in preterm milk	[78]
LST	mother age	↓ with age	[43,47,74]
LSTb	gestational age	↑ in preterm milk	[81,82]
LSTc	race/ethnicity	↑ in Asian mothers	[47]
	lactational stage	↓ over lactation	[32,49,65,66,69]
LNFP-I	genetic	↓ in non-secretors	[32,33,34,35]
LNFP-II	genetic	absent in some Lewis-positive individuals	[32]
	gestational age	negative association with gestational age	[74]
LNFP-III	genetic	found in Lewis-negative (Le−) individuals	[36,37]
	race/ethnicity	↑ in Swedish and Chinese mothers	[43,55]
	parity	↑ in primiparous mothers	[43,46,47]
	mode of delivery	↑ in natural deliveries	[55]
LNFP-IV	gestational age	negative association with gestational age	[74]
DSLNH	mother age	↓ with age	[43,47,74]
	maternal diet	↑ with multivitamin intake	[47]
DSLNT	gestational age	↑ in preterm milk	[81,82]
DFLNH, FLNH	mother age	↑ with age	[43,74]
FDSLNH	breastfeeding frequency	↑ with breastfeeding duration	[47,84,85]
TFLNH-II, IFLNH-I, DFpLNH-I, DFpLNH-II	gestational age	negative association with gestational age	[74]
Fucosylated HMOs (as a whole group)	race/ethnicity	↓ in non-secretor mothers from Malawi and Gambia (region dependent)	[50,51]
	maternal diet	↑ with vitamin B intake	[89]
Sialylated HMOs (as a whole group)	race/ethnicity	↓ in non-secretor mothers from Malawi and Gambia (probably connected with malnutrition)	[50,51]
	maternal diet	↓ with malnutrition ↓ with a lipid-rich diet ↑ with vitamin A intake ↑ fruit intake	[50,85,86,87] [90,91,92] [90]
	gestational age	↑ in preterm milk	[81,82]
General HMOs	maternal age	↑ with meat and poultry consumption	[95]

Increased levels are marked as ↑, decreased as ↓. * 2’-FL, 2’-fucosyllactose; 3’-FL, 3-fucosyllactose; 3’-SL, 3’-sialyllactose; 6’-SL, 6’-sialyllactose; 6’-GL, 6′-galactosyllactose; LNT, lacto*-N-*tetraose; LNnT, lacto*-N-*neotetraose; LST, sialyllacto*-N-*tetraose; LNFP, lacto*-N-*fucopentaose; DSLNH, disialyllacto*-N-*hexaose; DSLNT, disialyllacto*-N-*tetraose; DFLNH, difucosyllacto*-N-*hexaose; FLNH, fucosyllacto*-N-*hexaose; FDSLNH, fucosyl-disialyllacto*-N-*hexose; TFLNH-II, trifucosyllacto*-N-*hexaose-II; IFLNH-I, isomer 1-fucosyl-paralacto*-N-*hexaose-I; DFpLNH-I, Difucosyl-para-lacto*-N-*hexaose-I.
